# Supplementary figures and images for: Ascension of Chlamydia is moderated by uterine peristalsis and the neutrophil response to infection
Source: PLoS Comput Biol. 2021 Sep 7;17(9):e1009365. doi: 10.1371/journal.pcbi.1009365 (PMC8448331; doi:10.1371/journal.pcbi.1009365)

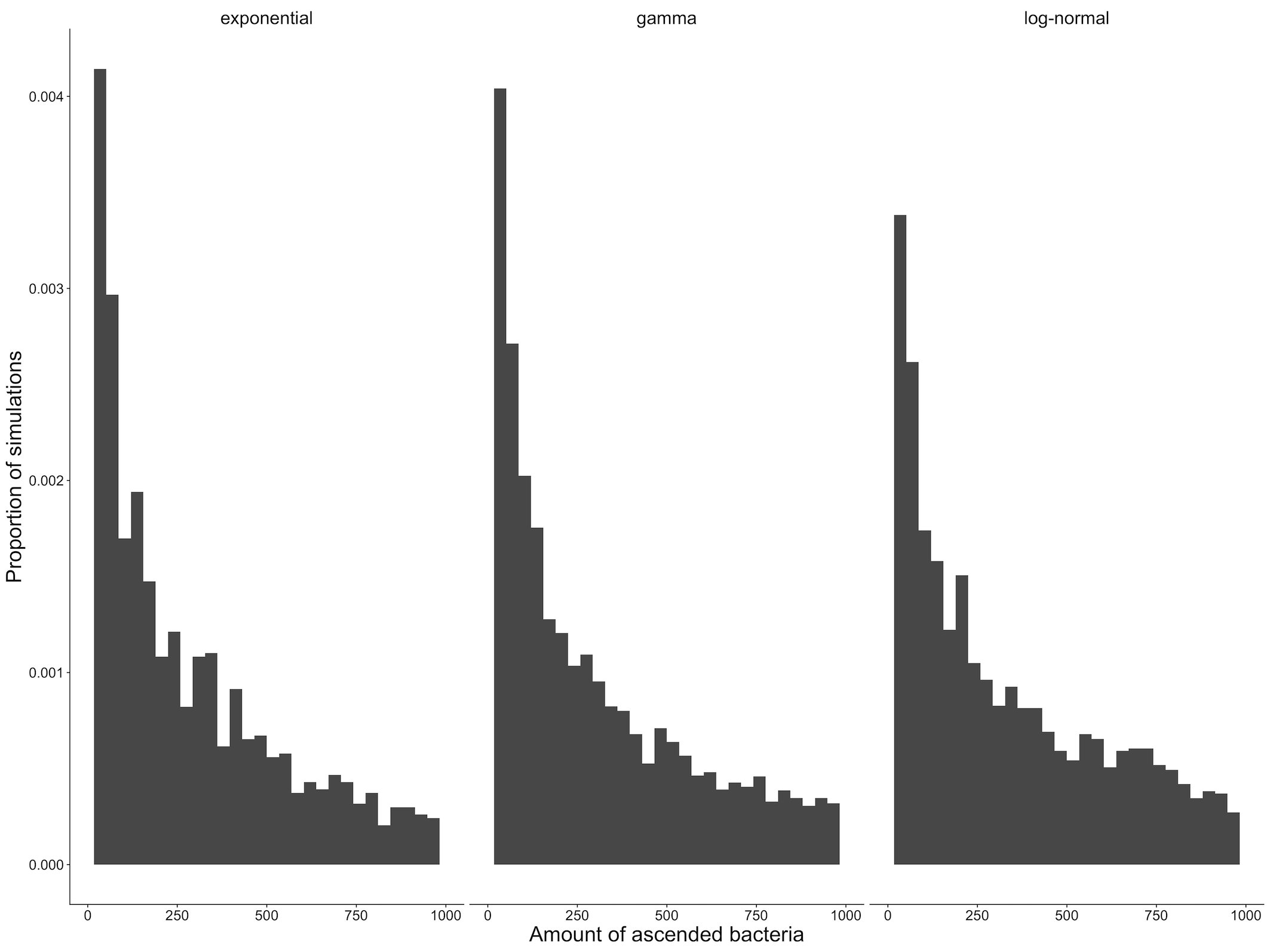

Supplement: S1 Fig — Each histogram represents 12,625 simulations from the model, where the underlying distribution for the rate parameters has been selected from an exponential distribution, a gamma distribution and a log-normal distribution. All distributions have identical means of 1/100. (TIF) [file pcbi.1009365.s001.tif]
