# Supplementary material for: Ascension of Chlamydia is moderated by uterine peristalsis and the neutrophil response to infection
Source: PLoS Comput Biol. 2021 Sep 7;17(9):e1009365. doi: 10.1371/journal.pcbi.1009365 (PMC8448331; doi:10.1371/journal.pcbi.1009365)
Supplement: S1 Text — (DOCX) [file pcbi.1009365.s002.docx]

**Supplementary materials**

*Parametrisation of key distributions*

The gamma distribution used to draw key rates for the model is parameterised in terms of a shape parameter $\alpha$ and a rate parameter $\beta$, such that the expected value is $\alpha/\beta$, and the probability density function can be represented as

$$f\left( x \right)=\frac{\beta^{\alpha}x^{\alpha-1}e^{-\beta x}}{\Gamma\left( \alpha\right)}$$

The negative binomial distribution, used for some key parameters of the model, is parameterised in terms of the number of trials before the n^th^ success, $k$ and the probability of success on each trial, p. The probability mass function is

$$f\left( x \right)=\binom{x + k - 1}{k - 1}\left( 1-p \right)^{k}p^{x}$$

The truncated gamma distribution referred to is similar to the gamma distribution, with the inclusion of a threshold value t, such that the support of the distribution is $\left( t,\infty\right]$.

**S1 Fig. A histogram of the amount of ascended bacteria under different distributional assumptions.** Each histogram represents 12,625 simulations from the model, where the underlying distribution for the rate parameters has been selected from an exponential distribution, a gamma distribution and a log-normal distribution. All distributions have identical means of 1/100.
