# Supplementary material for: Ascension of Chlamydia is moderated by uterine peristalsis and the neutrophil response to infection
Source: PLoS Comput Biol. 2021 Sep 7;17(9):e1009365. doi: 10.1371/journal.pcbi.1009365 (PMC8448331; doi:10.1371/journal.pcbi.1009365)
Supplement: Attachment — Submitted filename: ascensions_response_to_reviewers.docx [file pcbi.1009365.s003.docx]

We appreciate the opportunity to submit a revised draft of the manuscript entitled “**Ascension of Chlamydia is moderated by uterine peristalsis and the neutrophil response to infection**.” We thank the anonymous reviewers for their time and effort in providing feedback to our work. We have incorporated or addressed the changes suggested by the reviewers, which are highlighted below with our responses to the reviewer’s comments. All line numbers refer to the revised manuscript with tracked changes.

Reviewer #1: This is an interesting and well written paper on several factors that might moderate ascension of Chlamydia trachomatis infections to the upper genital tract in women. Results of the study indicate that infectious load, menstrual cycle timing, and the neutrophil response are important factors in the ascension of chlamydia. Results might increase insight in the pathology of chlamydia infections and factors might enable specific testing for at-risk of high risk women, or aid the development of interventions at timing of infection. In light of the current paradigm of chlamydia control and the question “what should we do with chlamydia control?” this paper is relevant and contributes to finding new ways of identifying high risk women.

Specific comments/questions include:
1. Line 67: In the introduction, several factors were mentioned to moderate in the ascension of chlamydia (menstrual cycle, stage of infection, immune status, genotype factors (host and pathogen) and infectious load). But not all were elucidated further in the introduction (for example genotype factors), while all factors were included in the model.***Author’s response****: Some of the factors noted in the introduction are not explicitly modelled, although we still believe these to be potentially influential. These were not included so as to make the model as parsimonious as possible. We have changed the wording in the introduction to make this clearer (Line 72-74)..*

2. Line 114: “The PMN response was observed to result in detached superficial epithelia from the mucosa, including infected cells and elementary bodies that remained healthy and intact during the process. It was suggested by Rank et al. that this response was a defensive mechanism from the perspective of the host organism in that it allows for infected cells that have been ejected from the epithelium to be cleared from the infection site. Further, it was proposed that from the perspective of the Chlamydia, this process allowed for movement away from infected sites where there is a lack of susceptible uninfected cells, transporting to new infection sites via fluid dynamics or tissue movement.” Wouldn’t you expect that, primarily the fluid dynamics or vaginal secretion, will descend the infected cells rather than ascend? How does that interreact?

***Author’s response:*** *The fluid dynamics caused by peristaltic contractions can both descend and ascend infected cells, as indicated by the sonography study of Kunz et al. that shows the peristaltic direction and intensity varies with the menstrual cycle. This behaviour is already present in the model (Lines 356 – 357). We have modified the introduction (Line 134-141) to better articulate this behaviour.*

3. Line 144: “Elucidating the processes involved in chlamydial ascension will provide a better understanding of the pathogenesis of Chlamydia associated PID and infertility. In turn, the moderating factors could enable more specific testing for at-risk women, or development of other interventions when infections are diagnosed.” Although I agree that elucidating processes around the ascension of chlamydia is relevant, specific testing for at-risk women concerning the menstrual cycle requires the need of timing of infection. Even more so the timing of ascending of the bacteria. How long might an infection reside in the lower genital tract before ascending?

***Author’s response:*** *In our model we have assumed that peristaltic contractions follow the menstrual cycle in direction and intensity, as per the vaginal sonography of Kunz et al. if peristalsis is not moving infectious material up the cervical canal, then peristaltic contractions will be moving material away from the lower genital tract, or there will be a period when peristaltic contractions are absent. An infection could conceivably reside in the lower genital tract for a number of days before ascension, if the migration event occurred at a time of minimal peristaltic movement. This is at the heart of what we explore with this model, and we have expanded the results sections text to clarify how we have explored the impact of timing relative to the menstrual cycle on ascension. We have also added a comment on line 145 to clarify that this is what we are exploring in the model and note the potential importance of burden at this point.*

4. Line 182: Is it correct that you assume that ejected infected cells contain viable RBs that will differentiate back in EBs? In other words that neutrophils were unable to inactivate chlamydia?

***Author’s response:*** *We refer to the study Rank et al. the describes that infected cells and EBs, once dislodged from epithelial layer remain viable as a potential means of transmission to new infection sites. There is likely to be death as an outcome of this event as well as availability of live chlamydia for transmission to new sites. The model incorporates the potential for death as a outcome across each of the states of chlamydia (intra or extracellular) as well as the potential for ascension. In order to clarify each of these parameters, in response to other comments as well, we have added the new parameter table (Table 1).*

5. Line 348: “However, increasing the rate at which extracellular bacteria infect target cells does appear to increase the chances of ascension. This points towards the increased utility of infected cells to the infection, as they influence migration and will produce greater amounts of bacteria in the future.” How would the rate at which EB infect target cells be increased? Can you elaborate more on how this points towards the increased utility of infected cells?

***Author’s response:*** *According to the assumptions we have made in this model, increasing the rate at which EB infect new cells increased the chances of ascension in two ways, by increasing the number of infected cells which continues the infection and influences ascension in the future, and by increasing the neutrophil response to more infected cells which in turn influences migration and therefore ascension. We have removed this statement from the results, but the point of the role in infected cells has been explored in the revised discussion text.*

6. Line 372: “The relationship between infectious load and the menstrual cycle further complicates matters. Infections with smaller infectious loads experience linearly decreasing chances of ascension as the menstrual cycle progresses, so an infection that occurs later in the cycle may have reduced chances of ascending. However, the opposite applies to high infectious loads, where infections have a higher chance of ascending as the menstrual cycle progresses closer to peristaltic movement in the cervico-fundal direction.” It seems that the chance of an ascending infection increases when you have either low initial loads early in the menstrual cycle or high loads later in the menstrual cycle. Are these chances comparable? Or is the chance of ascending with higher loads but later in the menstrual cycles still significantly lower compared to low initial loads early in the menstrual cycle. The conclusion in the abstract would suggest the latter.

***Author’s response:*** *According to our simulations, infections with the lower initial loads of 101 had a 48% chance of ascension if the infection began 30 hours prior to the onset of upwards peristaltic contractions, a 50% of ascension of infections beginning 60 hours prior and 38% for infections at 90 hours prior. By comparison, a high initial load of 10001 had a broadly constant chance of ascension between 39-43%, for infections that were initiated 22 or more days prior to upwards peristaltic movement.*

*Some of the results may be driven by the stochasticity present in the model, so we have attempted to robustly present these findings in Fig. 6. It would indicate that overall, lower initial loads have the highest overall chances of ascension. We have re-written on the results section to talk through the outcomes of the model and how they are influenced by the parameters.*

*This section of the results has been re-written (line 419 onwards):*
Overall, initial infectious load was influential on ascension events. Specifically, in this model we found initial loads in the range of 1000-3500 were most likely to ascend, with an apparent decrease in ascension found for higher loads (Fig 6A). The timing of initial infection relative to the onset of peristaltic contractions in the cervico-fundal direction (which is moderated by the menstrual cycle) had a much more marked impact on the simulated chances of ascension (Fig 6B). For example, infections that were initiated 30 hours prior to the onset of upwards peristaltic movement had a 7.8% of ascension, whereas infections initiated 10 days or more prior had a 42% of ascension. The relationship between infectious load and timing was also found to be critical in influencing ascension. Infections with smaller infectious loads experience linearly decreasing chances of ascension as the menstrual cycle progresses, so an infection that occurs later in the cycle may have reduced chances of ascending. However, the opposite applies to high infectious loads, where infections have a higher chance of ascending as the menstrual cycle progresses closer to peristaltic movement in the cervico-fundal direction (Fig 6C). Although a high initial load of 10001 had a broadly constant chance of ascension between 39-43%, for infections that were initiated 22 or more days prior to upwards peristaltic movement.

7. Furthermore the interaction between infectious load and menstrual cycle: Is that based on the menstrual cycle during time of initial infection? “Infections with smaller infectious loads experience linearly decreasing chances of ascension as the menstrual cycle progresses”, but if the infection is not cleared it might produce enough material so that in the following menstrual cycle there would be enough material that might cause a new infection in the upper genital tract? For example after one year after initial infection, without treatment, only 50% of women have cleared the infection. Could you elaborate on that?

***Author’s response:*** *In order to simplify the processes being studied for the model, we have assumed that infections in subsequent menstrual cycles are equivalent to new infections with the same infectious load. For example, an infection in month two of 1000 EBs is equivalent to a new infection of 1000 EBs. This assumption obviously does not account for the change in immune system response over a prolonged or persistent infection.*

*A richer model would look at the described ejection of infectious material due to the neutrophil response, and perhaps incorporate knowledge about movement of extrusions, to describe how movement of infected cells and chlamydia to new infectious sites may prolong the duration of an infection.*

*We have synthesised the limitations section of the discussion and added a sentence to declare that this model does not look at longer time frames, which would be important ot consider the longer time course of the infection (line 626-630).*

8. I’m am somewhat confused about how the neutrophil response relates to high and low initial infectious loads. In the introduction it was said that in case of high infection loads the immune response was sufficient to prevent ascension but not for lower loads. But in the results it was found that infections with high neutrophil responses were more likely to be one that ascend. Could you elaborate on that?

***Author’s response:*** *The referenced study in the introduction by Maxion et al. indicates that in ascension was more likely for lower infectious loads rather than higher ones, as the immune response for higher infectious loads usually prevented ascension. Our results agree with the observations in this study, in that lower infectious loads are more likely to ascend than higher ones (Figure 6). However, our results contradict the statement regarding the influence of the innate immune response in modulating ascension. We have assumed that the neutrophil response is critical in migrating cells from the epithelium to the cervical canal, and so the neutrophil response plays a different role in modulating ascension to that observed in the global innate immune system in the Maxion study. This point has been added to the discussion (Lines 787-790).*

9. Line 411: You propose to investigate the strict set of markers to investigate in a cohort study to examine development of upper genital tract pathology. How would you set up such a cohort?

***Author’s response:*** *We would propose a longitudinal cohort of women at-risk of infection, and measure the key markers proposed in this study (infectious load and an influx of neutrophils) at various stages of the menstrual cycle. To alleviate sample size issues, strong prior information could be incorporated from modelling studies (such as this one). However, in accordance with reviewer’s 2 suggestion we have streamlined the wording of the discussion and this point is now removed..*

10. Line 477: A study from Korn concluded that he proliferative phase of the menstrual cycle seems to be the primary risk factor for ascending infection by organisms associated with pelvic inflammatory disease. From the results of the study I don’t think that it can be concluded that timing of biopsy is equal to timing of ascension. The ascension might have happened days or weeks before and might differ between hosts.

***Author’s response:*** *This is a good point, and the discussion has been modified to reflect the fact that the timing of biopsy and ascension are distinct (Line 629). The observations of the Korn et al. study indicate the influence of the menstrual cycle on ascending infections, even if the timing of ascension was not measured exactly, which agrees with the broad findings of our study.*

Reviewer #2: Manuscript Number: PCOMPBIOL-D-20-02253
Title: Ascension of chlamydia is moderated by uterine peristalsis and the neutrophil response to infection

In this study, the authors constructed a stochastic model that describes the dynamics between extracellular chlamydial elementary bodies and infected cells containing chlamydial reticulate bodies in the epithelium and the probability that these ascend to the cervical canal. It is investigated how factors such as the infectious load, the menstrual cycle timing, and the neutrophil response could affect chlamydial ascension in women.

This is a very interesting study. Although it is clear that ascension can occur, it is unclear which factors facilitate or hinder ascension. The authors have developed a simple model that captures the main elements of this process, thus illustrating the role of some important factors, such as the infectious load or the phase of the menstrual cycle.

However, the study needs quite some improvement. The notation in the mathematical formulas is inconsistent and unclear and methods need some clarifications. English language needs improvement. References have to be added throughout the manuscript to support the arguments. The discussion contains too many repetitions, but does not cover important items, such as limitations and discussion/comparisons with similar studies. These points are explained in detail below.

**Authors’s responses:** We have edited the notation and formulas and worked throughout the text on the language.

My main consideration about the study is that, as the authors mention in the discussion (page 14), there are hardly any data to compare with model results for some level of validation. That makes the study kind of speculative or hypothetical. Also, the authors provide no evidence/reference to support their assumptions for the distributions of the parameters (e.g. Gamma, Beta, Negative Binomial and their means/shape parameters). If two or three of these parameters have a different distribution or the shape parameter is half of what the authors assumed, the results could be quite different. Although this is inherent for all models (input quality determines output quality), modelling studies nowadays provide extensive support for the model parameters (from earlier studies or directly estimated from data) AND model validation or fitting to data. That provides some level of confidence in the qualitative conclusions of modelling studies, which here lacks.

***Author’s response:*** *We thank the reviewer for their feedback. We appreciate that this study lacks any validation on external data, however we feel that due to the nature of the outcome being modelled, fitting the model to data would be difficult due to the inherent difficulties in observing ascension of chlamydia in women. A more reasonable approach to external validation of the modelling presented in this study would be to compare the results presented against Pelvic Inflammatory Disease (PID). This would require the inclusion in the model of the inflammatory processes that cause PID following an ascended infection. We felt that, as ascension has not been fully studied before in the literature, that this would distract from the intent of the study. However, external validation to empirical evidence of the results presented here would constitute interesting future work. We believe that this work will be a valuable contribution to the literature, as it elucidates a process that is difficult to study empirically.*

*We have addressed the feedback with regards to supporting our assumptions of the model in fuller detail below. The model includes stochastic terms which reflects randomness inherent in the process we are trying to study, and uncertainty about the values of parameters. We have also sampled hyperparameters for mean and shape values from distributions that reflect our uncertainty about these values. The randomness in the model, and the consistency of the outcome across a broad range of parameter values, gives us some confidence that the model results presented here are robust. Whilst the results presented here are speculative, they broadly correspond with previous empirical studies on the phenomena.*

Some additional points that could improve the study are listed below.

1. Line 187: In discussion, please explain how the results are affected by the assumption of instantaneous burst as opposed to continuous.

***Author’s response:*** *We have added a sentence to the discussion with regards to how this assumption would modify the observed extinction probability across different realisations of the model.*

2. Lines 215-223: The formula’s presented here are incomprehensible, especially since the notation in Figure 2 seems to imply that the rate is I*k1 or M1 * YC1 etc. The information in these formula’s is more or less repeated in the paragraphs from line 237 to 315. The authors could reorganize the text by deleting the repetitions and provide all information about each process in the model together (e.g. all formulas, explanations, assumptions, distributions, parameters about the immune response; then those about inclusion and bursts etc).

***Author’s response:*** *In order to better explain the formulation of our model, we have included a table of parameters for the model, including distributions, parameter values and supporting literature where appropriate. See new Table 1 in the manuscript and we have improved the mathematical notations throughout.*

3. Pages 8-9: Please remove Latex notation and use the same notation for each variable/parameter (e.g. gamma1 or gamma_1 in lines 267-270; or \gamma and Greek gamma in lines 307,308).

***Author’s response:*** *The notation in this section has been fixed to be consistent and readable.*

4. Lines 270-271: Gamma(1,00): what is after the comma?

***Author’s response:*** *This line has been fixed (Gamma1,100).*

5. Pages 8-9: Please provide some references or explanation for the assumptions about the distributions and the values of the parameters of the distributions used.

***Author’s response:*** *We have included a table of the assumptions and supporting literature for the parameters used in the study. Where a parameter is unique to our study, we have indicated as such in the table. Whilst many of the distributions were assumed, the model results appeared insensitive to the exact choice is most instances.We have included a Supplementary Figure and materials that demonstrates the impact of distributional choice on the amount of ascended bacteria.*

6. Lines 311-313: Ascension is defined as a yes/no event, depending on whether ascension occurred “when the simulation time finishes”. Maybe I have missed it, but when does the simulation finishes? How does the % ascending depend on the length of this time? How would the results about the proportion ascending differ with twice as long or half of this time?

***Author’s response:*** *Each simulation is begun with a fixed amount of time remaining before peristaltic contractions move fluid in the cervical canal upwards, causing any infectious material to ascend. We stop each simulation at the moment when peristalsis causes material to begin ascending, with the assumption that any material in the cervical canal at this point will ascend. This explanation has been added to the methods where the model is first described (line 189-193 in the revised manuscript.*

7. Legends of Figures 3-8: The legends discuss the results shown in the plots and draw conclusions. This is NOT the purpose of the legends. Please remove all text describing results and conclusions. The legends should explain what is shown in the plots and the scenarios, parameters, or methodology for each specific plot or result shown. Also, please add units where appropriate (e.g. “time” and “load” are measured in what?)

***Author’s response:*** *We have modified the legends of the figures to remove any discussion of the results and conclusions of the plots.*

8. Lines 323-332: These are not results of this study; please remove this paragraph.

***Author’s response:*** *This paragraph has been removed, and the explanation of the model has been moved and made more succinct Line 378-385.*

9. Lines 334-341: Please include reference to figure 3 at the beginning of the paragraph. The reader has to read the whole paragraph to understand where these results are shown. What is scenario A, B, C? do you mean plots A,B,C? What is the difference between the three plots? Are they just 3 realizations (with same parameter distributions and same initial conditions? If not, how do they differ? If they are with same parameters and initial conditions, then please add explicitly something like “plots A, B, C show three realizations of the same stochastic process with same parameter values and initial conditions showing the three major types of the course of the infection: not ascending, ejecting but finally dying out, surviving”.

***Author’s response:*** *This paragraph has been edited to give further clarity on the scenarios described in this paragraph, and how they are shown in the graphical figure. Line 362-380)*

10. Results: The whole section provides mostly a qualitative description of the results, e.g. this increases when that rates increase etc. Please provide some indicative quantitative results. For example, if the rate is 0.05, then the % ascending is 25%; with a rate of 0.10, the % ascending is 35% etc. Also, please remove conclusions, like “this points towards the increased utility of…”, and place them in the discussion.

***Author’s response:*** *We appreciate this feedback and agree. We have added in quantitative descriptions of the results as recommended, see Lines 459-460, 533-534, 561-562.*

11. Legend figure 4: “(A) increasing the rates of burst does not appear to impact the chances of ascension”: Again, this is description of result and drawing conclusions. The legend of this plot should be something like “The proportion ascending with different rates of burst”. Please make similar changes to all legends.

***Author’s response:*** *This text, and other figure legends with conclusions drawn, has been rephrased.*

12. Lines 356-358: This text is unclear. Please rephrase.

***Author’s response:*** *This paragraph has been rephrased to make the meaning more clear. (now line 402)*

13. Legend Figure 5: This plot is not a “summary of simulations”. Please use a proper name to describe this kind of figure. Please mention the number of simulations used for this plot and delete all conclusions.

***Author’s response:*** *We have modified the legend to refer to the histogram, included the number of simulations and deleted all conclusions in this figure legend.*

14. Legend Figure 6: The horizontal axis shows the “amount of time since infection”; please add units and change the legend since the phases of the cycle are not shown. The authors could add the phases.

***Author’s response:*** *The figure has been changed to include the units. We have also modified the figure legend to be explicit that the horizontal axis refers to the timing of infection (in minutes) in relation to the onset of peristaltic contractions in the cervico-fundal direction. Whilst this phenomenon is moderated by the menstrual cycle, we have left any speculation with regards to the role of specific phases of the menstrual cycle and ascension to the discussion section.*

15. Figure 7: Please explain/comment on the fact that the percentage of simulations ascending with high rates varies from zero to 100%! The legend for A reads “Innate immune system impact on EB death rate”, but I think it shows the impact of EB death rate due to innate immune system on % ascending”. The same for the other plots. Please update.

***Author’s response:*** *The results text for Figure 7 has been edited, line 448-460. Copied here* Our simulation experiments show that the chances of ascension are broadly independent of the immune systems response (defined as overall scale of response, Figure 7), indicated by death rate of infectious material (extracellular EBs and infected cells) and moderated by the global immune response (separated into innate and adaptive systems). It is important to note that these phenomena represent the relative strength and weaknesses of the overall immune system and its responses between hosts. The model predicts that increasing or decreasing the strength of the host immune response, that determines the removal of EBs and infected cells, would have no impact on the chances of a particular infection ascending. The outcomes of the model under these parameters was that death of infected cells (Figure 7B, D) at a higher rate (rather than EBs, Figure 7A, C) was more likely to reduce the proportion of infections that ascended in the simulations (although the outcomes of the model at high death rates were highly variable 0-100% of ascensions).

*The legends for figure 7 have been updated to better reflect the model output being observed. In order to best elucidate the impact of certain parameter values and model choices (including stochastic elements) on the model outcomes, we simulated over a large range of parameter values.*

16. Discussion: This section is quite long and contains a lot of repetition from introduction and results. Please remove repetitions. Please add a discussion of limitations of the study (e.g. impact of assumed distributions and parameters of the distributions), as well as comparison with earlier studies on the subject.

***Author’s response:*** *We have expanded upon a paragraph that discusses, in greater detail, some of the limitations of this study including the assumptions of the model. Overall, we feel that our results broadly match what has been described on ascension and the development of tubal pathology. This gives us some confidence that the results observed are not solely an artefact of specific model choices (eg. Parameter values, distributions). For example, the model replicates the observations of Maxion et al. in describing the interplay between initial infectious load and ascension. The long tail in the count of ascended bacteria (Fig 5.) agrees with the low rate of pathology development as seen in Oakeshott et al. The influence of timing of the infection, with respect to the menstrual cycle, coincides with the observations of Korn et al. We have edited the discussion considerably to tighten and focus the points made to the major outcomes of the model and how they sit in the context of biological and clinical data.*
