# Supplementary material for: Ascension of Chlamydia is moderated by uterine peristalsis and the neutrophil response to infection
Source: PLoS Comput Biol. 2021 Sep 7;17(9):e1009365. doi: 10.1371/journal.pcbi.1009365 (PMC8448331; doi:10.1371/journal.pcbi.1009365)
Supplement: Attachment — Submitted filename: ascension_response_to_reviewers_R2.docx [file pcbi.1009365.s004.docx]

We appreciate the opportunity to revise our manuscript entitled “**Ascension of Chlamydia is moderated by uterine peristalsis and the neutrophil response to infection**.” We thank the reviewers for their ongoing feedback on this work. We have incorporated changes in response to specific feedback from the reviewers, and our response are included in the comments below. All line numbers refer to the revised manuscript with track changes.

**Reviewer #1:** Following the first peer-review round, the authors made significant improvements to the manuscript. Elaborations and explanations have been made and the manuscript is more concise, specifically the discussion section.

Specific comments:
**1.** Table 1 is an improvement and gives insight in the parameters. The table itself is rather large, which decreases readability. Could you make the table more concise (for example Colom wide modifications).

**Response**

Table 1: We have reduced the size of the text for the ‘Meaning’ and ‘Supporting Literature’ heading, and corrected a few typographical errors in the ‘Value’ column. This has assisted with the readability of the table.

**2.** Headings in the result section come across as conclusions, for example line 393 and 413-414.

**Response**

We have modified the headings of the results to make it clear that the headings refers to our observations, and are not conclusion. On Line 400, the heading now reads

‘**Modelling indicates a role for initial infectious load and timing relative to the menstrual cycle for ascension’**

and the heading on Line 421-22 now reads

‘**Ascension chances are dependent on the neutrophil response in this model**

**3.** The discussion starts off with a limitation and comparison of the literature on line 443-451; commenting on dysfunctions of the peristaltic mechanisms, this comes out of nowhere. It might be worthwhile to mention it somewhere in the discussion, but please start the discussion focusing on the results of studied factors that influence chlamydial ascension

**Response**

The discussion has been reordered so that it begins with a comment on the impact of infectious burden (Line 484), and the paragraph on peristaltic dysfunction has been moved to line 518.

.
4. Line 525 please choose either examining/evaluating.

**Response**

This line has been reworded – see below in response to (5.).

**5.** In the conclusion you mentioned you could consider to further examining neutrophil levels as predictive markers for pathology development, but you also propose to that neutrophil levels could be useful as early indicators for pathology likelihood. The way that this is phrased weakens the conclusion. What is it you want to conclude? Could neutrophil levels potentially be useful as early indicators and therefor further examination is needed? Or is it useful even without further examination?

**Response**

We have reworded these sentences slightly to make the conclusion more clear. We believe that our simulation results indicate the neutrophil levels could be useful as early indicators of pathology, and so more examination is needed to validate this. This sentence has now been re-worded to state:

Line 558-561 ‘From our simulation results, we propose that cervical neutrophil responses and infectious burden could be useful as early indicators of the likelihood of pathology. This would need to be validated with further examination of neutrophil levels as predictive markers for pathology development of women who have recent exposure to *Chlamydia trachomatis.’*

**Reviewer #2: PCOMPBIOL-D-20-02253R1**
The manuscript is considerably improved and the authors have addressed most of my questions. However, there are some points that need some clarification/improvement:
**1.** How much time were the simulations carried out to determine whether ascension occurred or not? Did the authors examine/assess whether the % ascending depends on the duration of simulations? I had raised this point in my first review (point 6), but the response of the authors was unclear: they write that the “simulation is stopped when peristalsis causes material to begin ascending”. In some simulations, ascension does not occur, so my question is: how long were these simulations carried out? Did the authors assume that all infections end with either 0 bacterial load or ascension and continued the simulations until one of the two events occurred? In that case, what was the max time needed to reach one of the two endpoints? That helps to understand the time scales. Please add this information and explanation in the main text.

**Response**

The stage of the menstrual cycle was measured in the number of minutes before peristaltic contractions began in the cervico-fundal direction. Peristaltic contractions in this direction would initiate regardless of the presence of infectious material, however if material was present then ascension was said to have occurred. This follows from the paragraph in the introduction (Line 130-138) that discusses the bi-directionality of uterine peristalsis, as shown in work by Kunz and Leyendecker (2001).

In our simulations, we modelled over a range of times from 0 to 43,200 minutes in steps of 1800, with 10 simulations per value, across a grid of initial conditions for infectious loads. This is reflected in **Lines 272-274,** with the following sentence added to further clarify this point:

‘The longest simulation was therefore 43,200 minutes long. A simulation either ended with material ascended, 0 bacterial load or with some infection present in the epithelium but not in the cervical canal.’

**2.** A point relating to time: in the author’s response to point 7 of Reviewer 1, the authors state that “an infection in month 2 of 1000 EBs is equivalent to a new infection of 1000 EBs”. However, an infection of 1000 EBs in month X, may become an infection of 2000 EBs (for example) in month Y – and that possibly depends on the time Y-X? Can you explain how that was modelled? If infections in subsequent menstrual cycles are equivalent to new infections, how was the menstrual cycle included in the model?

**Response**

As noted in our previous response, the length of time of the simulation (representing the stage of the menstrual cycle as the menstrual cycle determines uterine peristalsis) was chosen as an initial condition of each simulation.

In order to simplify the model, we only modelled the first period of the infection until the end of the first menstrual cycle. However, as noted by Reviewer 1, many infections do persist beyond this time frame, in many cases up until 12 months. Our results may be extrapolated to determine the chances of ascension in subsequent months after the first one, although a richer model specifically developed to answer this question may be more appropriate.

Line 539-542 this sentence has been added to the discussion

The results may be extrapolated to determine the chances in subsequent cycles after the initial cycle, although a model specifically developed to answer the likelihood of ascension in subsequent months may be a more appropriate approach for longer range estimates.

Abstract: Further, we have also edited a sentence in the abstract to make the timeframe and scope of this model more clear to the reader.

'We constructed a mathematical model based on a set of stochastic dynamics to elucidate the moderating factors that might influence ascension of infections.'

 We changed this to

'We constructed a mathematical model based on a set of stochastic dynamics to elucidate the moderating factors that might influence ascension of infections in the first month of an infection.'

**3.** Lines 233-239: “chlamydia” usually refers to the disease itself. Please use here another term instead of chlamydia and, preferably, the same term you use in the previous paragraphs (EB, if I understand it correctly) to avoid confusion.

**Response**

We have modified the model description to refer precisely to the elementary bodies (EB) form of the organism. This has also been corrected throughout the text.

**4.** Fig.3: In line 372, it says that the three scenarios A, B, C show realizations of the same stochastic process with distinct parameter values and same initial conditions. In the legend and in the Results section, it is stated that the three scenarios A, B, C, start with different initial load. I would expect that the “initial load” is one of the initial conditions, not a parameter. What exactly is the initial load? Is it the value of one of the model variables at t=0 or one of the parameters and which one? Please give the actual initial load (not an approximation, like “~800 EBs”; and not only a comparison “more/less than in A”) for each of the scenarios A,B,C.

**Response**

We have included the values of the initial infectious loads in the legend for Figure 3. Infectious load is an initial condition of the model, for the amount of extracellular chlamydia in the epithelium at t=0 ($X_{C_{1}}$as per the model definition on 202).

In the realisations of the model in Figure 3, we modified the initial condition of infectious load, but not the initial conditions of time until peristaltic contraction in the cervico-fundal direction, and the initial amount of infected cells and ejected material.

We have edited the relevant results paragraph (Lines 361-3) to make this clearer.

**5.** The description of results from Fig.3 seems to imply the importance of the “initial load” for the outcome of ascension. That raises the following question: what was the initial load in the other results (Fig. 4-8) and how were these results affected by the initial load?

**Response**

The other results discussed simulated over a range of infectious loads (10 simulations at each value of infectious load from 1 to 10001 in steps of 100, see Line 274). The results in Figures 4 – 8 display simulations over this range of initial load. We have referenced the potential influence of infectious load in Figure 6(A), the interaction of infectious load and timing with respect to the menstrual cycle in Figure 6(C), and the interaction of infectious load and the neutrophil response in Figure 8(B). Further information has been added to the figure legends for figures 4, 5, 6, 7, and 8 to clarify this point.

**6.** Lines 397-398: It is nice that the authors added these percentages in the text. It is a pity, however, that these cannot be “seen” in the plot. Could the authors add more labels on the axes (e.g. showing values between 0 and 0.05 on the horizontal axes and values between 0 and 0.25 on the vertical axes) to increase readability of the plots.

**Response**

We have updated the labels on Figure 4 to make the percentages more readable. We have also done the same for Figure 8(A). New figures 4 and 8 are uploaded on the publisher system.

**7.** The authors use various terms for the disease chlamydia and the organism (C. trachomatis) causing the disease and sometimes use the same term for the disease and the organism. To avoid confusion, it is advisable to use distinct terms for the organism and the disease and to use these terms consistently throughout the manuscript.

**Response**

We have referred to the organism as *C. trachomatis* consistently throughout the manuscript to avoid any confusion (or were appropriate used the word pathogen). In the model definitions, figure legends and the main table of parameters, we have referred to the organism as extracellular chlamydia or elementary bodies where appropriate.
